# Smartphone Apps for Preventing Adolescent Health Problems Among Health Care Professionals: Systematic Search and Quality Assessment

**DOI:** 10.2196/83908

**Published:** 2026-09-11

**Authors:** Elodie Million, Marijn Kuijper, Carole Bautista, Lola Pirat, Bruno Falissard, Béatrice Lognos, François Carbonnel

**Affiliations:** 1 Department of General Practice, University of Montpellier, Campus Arnaud de Villeneuve, 641 Av. du Doyen Gaston Giraud, Montpellier, 34090, France, +33(0)662865261; 2 Desbrest Institute of Epidemiology and Public Health (IDESP), UMR UA11 INSERM, University of Montpellier, Montpellier, France; 3 CESP (Centre for Research in Epidemiology and Population Health, INSERM), University Paris Sarclay, Paris, France

**Keywords:** adolescent, primary care, preventive medicine, mobile apps, mental health, sexual health

## Abstract

**Background:**

Health care professionals must consider multiple dimensions of prevention when consulting with adolescents. Identifying risky behaviors early in adolescence is crucial for reducing both morbidity and mortality. General practitioners are increasingly eager to incorporate digital tools for prevention into their consultations with adolescents; however, the relevance and clinical validity of these digital tools are not always established or well-known. Consequently, primary care professionals require guidance and support in selecting relevant mobile health (mHealth) tools.

**Objective:**

The aim of this study is to identify relevant and useful digital apps to help primary care professionals detect at-risk adolescents across all recommended areas of prevention: orthopedics, mental health, substance abuse, risk behaviors, sexual health, vaccinations, social relationships, and nutrition.

**Methods:**

A systematic review of smartphone apps, with an analysis of content quality, was carried out by 4 researchers using the PRISMA (Preferred Reporting Items for Systematic Reviews and Meta-Analyses) checklist. The App Store and Google Play Store platforms were surveyed. The inclusion criteria were as follows: free of charge, date of last update, availability in French or English, relevance of the preventive approach to adolescents, and scientific validation. Four health care professionals assessed the apps: 2 selected the apps relevant to health care professionals, then 3 analyzed these apps using the French version of the Mobile App Rating Scale (MARS-F). Intraclass correlation coefficient, model (2,1) (2-way random effects, absolute agreement, single measures); standard error of measurement; and mean absolute error were also calculated.

**Results:**

A total of 976 apps were identified, 49 of which had disappeared from the platforms prior to analysis. Nine apps were retained. Seven (0.72%) were included after evaluation using the MARS-F: 2 on mental health and 5 on sexual health (including 3 on contraception only). The mean MARS-F interrater score ranged from 2.5/5 to 3.8/5. The global MARS-F score demonstrated a pooled SD of 0.60 and an intraclass correlation coefficient (2,1) of 0.0003, resulting in a calculated standard error of measurement of 0.60. The average discrepancy between raters was a mean absolute error of 0.53.

**Conclusions:**

No similar studies have been identified in the literature that specifically focus on mobile apps designed to support health care professionals in delivering preventive care to adolescents. Of the 8 areas of prevention identified as relevant for adolescents, only 3 are addressed by the apps validated through our methodology (5 focus on sexual health). Consequently, current apps are insufficient to support health care professionals in their overall preventive work with adolescents. Such a review should be conducted systematically prior to the development of any new tool to prevent duplication and channel creative efforts toward truly innovative digital solutions. Furthermore, a thorough analysis of relevant, recommended websites is essential, as these resources complement the use of mobile apps designed for health care professionals.

## Introduction

Adolescence is a period of transition from childhood to adulthood. Adolescents are subject to many biopsychosocial changes, including the desire for autonomy and experimentation, the need to identify with peers, and risk-taking [1-6]. Health care professionals must consider a wide range of preventive domains during consultations with adolescents: sexual health, mental health, substance abuse and addiction, diet, physical activity, orthopedic disorders, vaccinations, screen use, and social relations [6-11]. Preventive actions are difficult for health care professionals to implement because of the complexity of consultations with adolescents [10-15]. Early identification of risky behaviors in adolescents is essential for reducing morbidity and mortality [11,16]. Although various recommendations and predominantly nondigital tools exist to support adolescent preventive care during consultations [8,9,14,17,18], health care professionals often find them difficult to implement effectively in practice.

The World Health Organization (WHO) defines eHealth as “digital services for human well-being,” that is, the application of information and communication technologies to health and well-being [19,20]. Mobile health (mHealth) covers medical and public health practices based on mobile devices (mobile phones, patient monitoring systems, personal digital assistants, and other wireless devices) [19,21]. The development of health-focused websites and smartphone apps has contributed significantly to improving both the quality of care and patient health outcomes [20]. Digital tools are used in routine care by health care professionals and patients. In 2021, over 327,000 mHealth apps were listed in digital stores [21]. French general practitioners generally view mHealth apps and devices favorably and express readiness to integrate them into their clinical workflows [15,22]. In a qualitative study carried out among French general practitioners, the need for a preventive tool in consultations with adolescents was identified, with a preference for a practical digital tool [15]. Despite the desire of general practitioners to use these digital tools, the relevance of these tools is not always established or known [15,17].

Many apps are easy to use during consultations, and general practitioners often do not have the skills or time to select from the wide range on offer. General practitioners need guidance to choose relevant apps. The main aim of this study was to identify relevant and useful digital apps to help primary care professionals detect at-risk adolescents across all recommended areas of prevention: orthopedics, mental health, substance abuse, risk behaviors, sexual health, vaccinations, social relationships, and nutrition.

## Methods

### Study Design

A systematic review of smartphone apps with an analysis of content quality was carried out by 4 researchers. One general practitioner resident (CB) and 1 experienced general practitioner (EM) carried out the search for apps on search engines and the initial selection. Two general practitioner residents (MK and LP) and 1 experienced general practitioner (EM) evaluated the apps using the French version of the Mobile App Rating Scale (MARS-F) [23]. The study protocol was designed following the PRISMA (Preferred Reporting Items for Systematic Reviews and Meta-Analyses) guidelines (items not relevant to a systematic search of apps were considered not applicable; Checklist 1) and the PRISMA-S (Preferred Reporting Items for Systematic Reviews and Meta-Analyses Literature Search Extension) guidelines (Checklist 2) [24,25]. We took into account the specialized reporting items suggested by the PASSR (Protocol for App Store Systematic Reviews) guidelines (Checklist 3) to address the unique challenges of searching and analyzing mobile app stores [26]. The complete and detailed study protocol is provided in Multimedia Appendix 1.

### Search Strategy

A systematic search was performed on the 2 major digital platforms: the Apple App Store and the Google Play Store. These platforms are used by approximately 99% of smartphone users to download their apps [27]. Two similar searches, using the same method, were conducted between August 2022 and March 2023, and then between March 2023 and May 2023. The platforms were queried in French and English using keywords related to adolescent prevention areas derived from MeSH terms [28]. MeSH terms were preidentified in a literature review according to prevention themes (Table 1). MeSH terms were replaced with non-MeSH synonyms when a search did not find any relevant results. The choice of words was discussed between the 2 researchers, CB and EM. Each keyword was used individually on the app platforms. CB and EM carried out the search on an iPhone 11 smartphone running iOS 15 (Apple), an ASUS VivoBook computer (ASUSTeK Computer Inc), and a Google Pixel 7 smartphone with the S91xBXXS1AWD1 update. The search ended when the search engines no longer provided new apps: keywords were entered into the search tool, followed by an exhaustive review of the descriptions of the apps offered.

**Table 1.** English keywords.

Prevention areas	English keywords
Orthopedics	Kyphosis Spinal curvatures Vertebral body Lordosis Spinal or vertebral column Scoliosis Back pain Osgood-Schlatter disease^a^ Osteochondrosis Scheuermann disease^a^ Patellofemoral pain syndrome
Substance abuse, screens, and addictions	Alcohol Nicotiana Drug Cannabis Heroin prevention^b^ Cocaine Ecstasy Screen time
Mental health	Sleep-wake disorders Anxiety Depression Suicide Attention-deficit/hyperactivity disorder Psychosis Autism
Nutrition	Obesity Deficiency diseases Feeding and eating disorders
Sexual health and puberty	Sexually transmitted infection Unsafe sex Contraception Puberty
Social relations and violence	School dropout Harassment, bullying^a^ Friend Violence
Vaccinations	Vaccination

a Non-MeSH words.

b The term “heroin” was not retained because the search results yielded only games.

### Selection Process and Eligibility Criteria

The selection by EM and CB followed a 2-stage process. First, EM and CB screened the apps based on their titles, descriptions, and metadata provided in the app stores. Second, the shortlisted apps were downloaded and tested for full eligibility. The initial selection of apps was based on inclusion and exclusion criteria from digital platforms. In cases of doubt about an exclusion criterion, the decision was made through discussions between CB and EM.

To meet our inclusion criteria, the apps had to (1) relate to one of the selected areas of prevention (orthopedics, mental health, substance abuse, risk behaviors, sexual health, vaccinations, social relations, nutrition); (2) be relevant to facilitating the preventive approach of health care professionals during consultations with adolescents; (3) be available in French or English; (4) have been scientifically validated (developed by health care professionals or a learned medical society and/or based on scientific or government recommendations); (5) be free (apps free for only a trial period were not selected); (6) have been updated within the 3 years preceding the end of the search, at least by 2020 (since 2022, the Google Play Store has removed apps that have not been updated for more than 2 years [29], and for the App Store, this period is 3 years for apps with few downloads in the last 12 months [30] but not all catalogs have yet been updated).

The exclusion criteria were as follows: (1) lack of information about the scientific data used to design the app’s content, (2) games without medicoeducational content, (3) wallpaper apps, (4) religious apps, (5) apps that offer links to medical appointments, and (6) advertising apps.

Inclusion criteria were based on the data available on each app’s presentation page: the title, description, images, and general information about the app. If any data were missing, the apps were opened to search for the missing information. Apps were excluded after download if they were no longer available in app stores at the time of evaluation by one of the evaluators. We chose to focus on free apps to ensure that the tools identified are accessible to all health care professionals, regardless of institutional funding or individual socioeconomic status, thereby promoting digital health equity regardless of which health care professional a patient consults [31]. Health care professionals have already invested in costly practice tools such as business software and telecare tools. We therefore wanted to offer them apps with no impact on their professional workloads. The high cost of an app is one of the main reasons why it is not downloaded [32,33]. Selecting free apps also limits the risk of commercial bias or conflicts of interest that can be associated with paid models.

As these digital tools are intended for use in clinical consultations, scientific rigor is nonnegotiable. Using unvalidated tools poses an ethical risk to the patient-provider relationship and the safety of care.

### Quality Appraisal and Data Extraction

MK, LP, and EM evaluated apps using the MARS-F [23]. The MARS and its French version (MARS-F) have been validated for the overall quality assessment of apps in the field of mHealth [23,34,35]. The app evaluations took place between December 2023 and January 2024.

The MARS-F assesses four objective quality dimensions: (1) Engagement (entertainment, interest, customization, interactivity, and target group), (2) Functionality (performance, ease of use, navigation, and gestural design), (3) Esthetics (layout, graphics, and visual appeal), and (4) Information (description accuracy, goals, information quality or quantity, visual information, credibility, and evidence base). A subjective quality score and an app-specific section were also proposed. Each item is rated on a Likert scale from 1 (“inadequate”) to 5 (“excellent”).

When specific objectives were not delineated and information was lacking, the respective items were rated as “not attributable” (NA). Consequently, these items were excluded from consideration in the overall scoring process. In total, MARS-F is a total score out of 5, corresponding to the average of these 4 sections. For each evaluator, an average for each section (A, B, C, and D) from 1 to 5 was calculated, followed by an interevaluator average for each section. An overall average of the interrater MARS-F score was used to evaluate the apps. To ensure the selected apps were suitable for clinical practice and met the needs of health care professionals working with adolescents, specific quality cut-off scores were applied during the screening phase. Apps were excluded if they failed to meet the following minimum thresholds across the MARS-F sections (Multimedia Appendix 1). Apps were excluded if MARS-F ratings were below 3 for target group relevance (Item 5, Section A), below 2 for all technical usability items (Section B: Functionality), or if ratings were insufficient within the Information Quality domain (Section D: Item 15 <2 or NA, Item 16 <2, Item 17 <4). During the quality appraisal, if an exclusion criterion was found by only 1 of the 2 evaluators for the final evaluation score, the third researcher (BL) was asked to discuss the results, and consensus was sought.

From the apps selected on digital platforms by EM and CB, 3 researchers took part in evaluating them: the 2 general practitioner residents (MK and LP) divided up the prevention themes for analysis, and the experienced general practitioner (EM) analyzed all the apps in a blinded manner. All 3 completed the MARS-F [23] separately. A common table was then filled based on these MARS-F results. To ensure rating consistency, LP and MK underwent a structured calibration process led by the veteran expert (EM). During the 2-session calibration process, informal reliability checks were conducted to ensure rater alignment. The 3 researchers independently scored a pilot sample of test applications. Discrepancies and borderline cases were thoroughly discussed until a 100% consensus was reached on all criteria. However, no formal statistical reliability metrics, such as a training intraclass correlation coefficient (ICC), were computed during this preliminary phase.” The training consisted of two 2-hour sessions. The first session involved a comprehensive review of each MARS-F item and its scoring criteria. In the second session, conducted 3 weeks later, the 3 researchers independently and blindly evaluated 2 apps randomly selected from the initial search results. Discrepancies were discussed collectively, and scoring rules were clarified under the supervision of the researcher EM. Throughout the formal evaluation period, raters had access to expert guidance to resolve technical queries. Specific apps were not discussed until the final consensus meeting to maintain blinded assessment integrity. While a formal training ICC was not calculated, this iterative calibration was designed to minimize subjective bias.

### Statistical Analysis

MARS-F scores were presented as means and SDs. To assess the reliability of the MARS-F ratings (global and subscales), we used a multimetric approach. Interrater reliability was calculated using the ICC, model (2,1) (2-way random effects, absolute agreement, single measures), along with the SD. To address measurement precision, we calculated the standard error of measurement (SEM) using the formula SEM=SD×√(1–ICC). Descriptive statistics, including the mean and SD, were generated for each app and for each global scale or subscale to characterize the distribution of quality ratings. We analyzed the expert’s ratings (EM) against the residents’ ratings (MK or LP). Since the ICC is a ratio of intersubject variance to total variance, the lack of variability between subjects in a small sample could mathematically deflate the ICC, even when raters are in close agreement. To provide a more accurate assessment of rater reliability, we also calculated the mean absolute error (MAE). The MAE represents the actual average point difference between raters on the 5-point scale.

An ICC above 0.75 was considered excellent, good between 0.60 and 0.74, fair between 0.40 and 0.59, and poor if below 0.40. We have applied an interpretation threshold for MAE: excellent (<0.5), good (0.5‐0.75), fair (0.75‐1.0), and poor (>1.0).

ICC analyses were performed using JASP (version 0.96; 2026; University of Amsterdam), and MAE, SD, mean, and SEM were calculated in Microsoft Excel.

### Ethical Considerations

A request for authorization has been made to the Data Protection Officer of the University of Montpellier. In addition, the study did not require authorization from the “*Comité de la Protection des Personnes*” (Personal Protection Committee), as it did not involve experimentation on individuals and did not fall within the scope of the General Data Protection Regulation and, therefore, of MR004. This study complies with the rules of the Helsinki Declaration [36].

## Results

### App Selection

**Figure 1.**
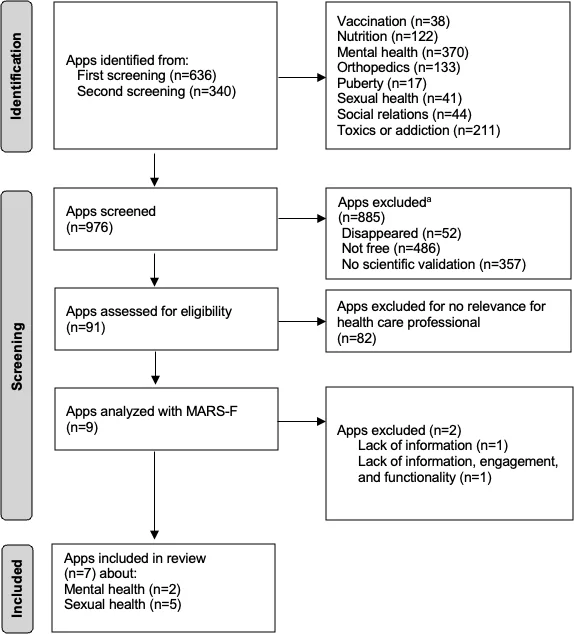
Flowchart. ^a^Apps can be excluded for several criteria. MARS-F: French version of the Mobile App Rating Scale.

Using the preidentified keywords, the double screening identified 976 apps. Forty-seven apps had already disappeared from the platforms between the 2 screenings and at the time of the first analysis by CB and EM; 2 more disappeared during the final evaluations by LP, MK, and EM, giving a total of 52 apps that had disappeared during the search. In the end, 9 apps were retained. After evaluation with the MARS-F, 7 were included (Figure 1). The mean MARS-F interrater score ranged from 2.5/5 to 3.8/5 (Table 2).

**Table 2.** MARS-F^a^ score for the 7 included apps.

App evaluated and rater	Section A	Section B	Section C	Section D	Average app score, mean (SD)	Interrater score, mean (SD)
*Agenda du sommeil antifatigue* ^b^						3.5 (0.14)
EM	2.4	4.7	3.3	4.1	3.6 (0.86)	
MK	2.6	4.7	2.6	3.5	3.3 (0.86)	
mhGAP-IG 2.0 App (e-mhGAP)						3.6 (0.25)
EM	2.6	4	3	4	3.4 (0.61)	
MK	3.8	4.5	3.3	4	3.9 (0.43)	
WHO^c^ Contraception Tool						3.8 (0.05)
EM	2.6	4.2	4.3	4.5	3.9 (0.75)	
LP	3.6	3.7	3.6	4	3.7 (0.16)	
Contraception Point-of-Care						2.5 (0.08)
EM	1.6	3.7	2	3.6	2.7 (0.93)	
LP	1.6	3.2	2.6	2.1	2.4 (0.59)	
Contraception Eligibility Tool						3.1 (0.15)
EM	2.6	4.5	3	4.2	3.5 (0.79)	
LP	2	3	3	3	2.7 (0.43)	
Practice Family Planning						3.0 (0.41)
EM	2.6	4.7	3.3	3.6	3.5 (0.75)	
LP	2.6	2.5	2	3.4	2.6 (0.50)	
Learn Family Planning						3.1 (0.47)
EM	2.6	4.5	3.3	4	3.6 (0.71)	
LP	2.6	2.5	2	3.4	2.6 (0.50)	

a MARS-F: French version of the Mobile App Rating Scale.

b App is available only in French.

c WHO: World Health Organization.

### Quality of Apps Based on MARS-F

The evaluation scores for the 9 evaluated apps were listed in a table (Multimedia Appendix 2), and a summary of the content of the apps was produced for the apps not excluded from the analysis (Multimedia Appendix 3). We provide a summary of the MARS-F scores for the 7 included apps (Table 2) and a short summary of these apps.

Some items in MARS-F have been rated NA. We describe the NA items present in the apps evaluated by MARS-F in Table 3.

**Table 3.** Summary of quotes^a^.

MARS-F^b^ item	*Agenda du sommeil antifatigue*	mhGAP-IG 2.0 App (e-mhGAP)	WHO^c^ Contraception Tool	Contraception Point-of-Care	Contraception Eligibility Tool	Practice Family Planning	Learn Family Planning	*Bariatrique Québec*	USPSTF Prevention TaskForce	NA^d^	NA, %
Item 14 (Goals)	0	1	1	0.5	1	1	1	0	0.5	6	66
Item 17 (Visual information)	0	0	1	0	1	0	0	0	0	2	22
Item 19 (Evidence base)	1	1	1	1	1	1	1	1	1	9	100

a “1” represents an NA rating by both evaluators independently, “0” represents no NA for this item, and “0.5” represents an NA rating by only 1 evaluator independently.

b MARS-F: French version of the Mobile App Rating Scale.

c WHO: World Health Organization.

d NA: not attributable.

### Reliability Evaluation

The global MARS-F score demonstrated a pooled SD of 0.60 and an ICC (2,1) of 0.0003, resulting in a calculated SEM of 0.60. The average discrepancy between raters remained low, with an MAE of 0.53. Across subscales, the SEM ranged from 0.41 (Engagement) to 0.79 (Functionality) (Table 4).

**Table 4.** Reliability table^a^.

Scale or subscale	ICC^b^ (95% CI)	Global SD	Min-Max	SEM^c^	MAE^d^
Global MARS-F^e^	0.0003 (−0.51 to 0.61)	0.60	2.55‐3.92	0.60	0.53
Engagement	0.54 (−0.12 to 0.88)	0.61	1.60‐3.80	0.41	0.4
Functionality	0.00 (−0.46 to 0.61)	0.79	2.50‐4.75	0.79	1.03
Esthetics	0.33 (−0.35 to 0.79)	0.70	1.66‐4.33	0.57	0.75
Information	0.26 (−0.15 to 0.72)	0.59	2.66‐4.50	0.51	0.66

a ICC (2.1) values are affected by range restriction (low intersubject variance) and low number of apps. MAE is provided to complement ICC analysis and clarify reliability.

b ICC: intraclass correlation coefficient.

c SEM: standard error of measurement

d MAE: mean absolute error.

e MARS-F: French version of the Mobile App Rating Scale.

The interrater reliability analysis comparing the veteran expert (EM) and general practitioner residents (MK and LP) yielded variable results across the MARS-F subscales (Table 4). As expected, the ICC (2,1) for the overall MARS-F score was poor at 0.0003 (95% CI −0.51 to 0.61). While ICC values for several subscales remained poor, ranging from 0.000 to 0.540, these findings are largely attributable to range restriction caused by the high homogeneity of app quality scores in our small sample (n=9). To account for this statistical artifact, we calculated an MAE to assess absolute agreement. The MAE for the global score was 0.53 (good), indicating that ratings differed by approximately half a point on the 5-point scale, which represents good clinical agreement.

### Description of the Apps

Of the 976 apps identified, 37.9% (ie, 370) and 21.6% (ie, 211) were found using mood-related keywords and toxic consumption-related keywords, respectively, compared with only 13.6% (ie, 133) and 3.9% (ie, 38) for orthopedics and vaccination. Nine apps were subjected to double-blind analysis using the MARS-F, and 7 were included. The “*Bariatrique Québec*” app was excluded from Section D (Information); the “USPSTF Prevention TaskForce” app was excluded from 3 sections: A (Engagement), B (Functionality), and D (Information). Among the 7 apps selected from the 976 apps identified (0.72%), 71.4% (ie, 5) were related to sexual health, all of these sexual health apps (100%) dealt with contraception, and 60% of these apps (ie, 3) dealt very briefly with sexually transmitted infections (1 dealt with antiretrovirals and 2 with condom use). About 28.6% of the selected apps (ie, 2) were relevant to mental health. No apps were selected for the following themes: vaccination, nutrition, orthopedics, puberty, social or toxic relationships, and addictions.

We summarized the 7 apps selected for use by health care professionals to support prevention initiatives during consultation with adolescents as follows:

- *Agenda du sommeil antifatigue*: this French-language app allows patients to maintain a digital “sleep diary” to better understand their sleep habits and sleep efficiency, and potentially improve their sleep hygiene. The app serves as a clinical tool for physicians, enabling them to evaluate adolescents’ sleep patterns, identify underlying sleep problems, and subsequently optimize the management of sleep disorders.
- mhGAP-IG 2.0 App: this app, available in several languages including English and French, is designed for health care providers around the world and is based on WHO recommendations. It serves as the electronic version of WHO’s mhGAP Intervention Guide, version 2.0, for mental, neurological, and substance use disorders in nonspecialized health settings. The app provides information on mental, neurological, and substance-related disorders. It offers detailed descriptions of the diseases, questionnaires for identification and/or assessment, and recommendations for treatment. This app is highly valuable to physicians for detecting mental disorders and self-harming behaviors in adolescents. However, while the app met the minimum threshold for inclusion based on the MARS-F criteria, certain nonfunctional features may negatively affect its overall usability in clinical practice.
- WHO Contraception Tool: developed by the WHO, this app helps health care professionals select the contraceptive method that is best suited to the patient’s medical history and characteristics and that complies with good-practice recommendations. Contraceptive methods are ranked from 1 to 4 according to the level of recommendation (1: “use possible,” 2: “use possible with conditions,” 3 and 4: “use not recommended”). This app uses the WHO 2015 recommendations and is available in several languages, including English and French. The app also allows providers to integrate patient preferences and delivers comprehensive information regarding emergency contraception, contraceptive efficacy, and antiretroviral drugs.
- Contraception Eligibility Tool: this app is the exclusive English version of the “WHO Contraception Tool” and is based on 2 WHO recommendations from 2015 to 2018. It has been developed for humanitarian professionals. It is a tool for selecting the recommended contraception according to the medical data and history of patients, with the same ranking key from 1 to 4 as the 2 previous apps. The app also provides additional clinical guidance on initiating contraception, infertility management, emergency contraception, contraceptive efficacy, the identification of victims of violence against women, and the management of missed pills.
- Contraception Point-of-Care: this app offers interactive clinical algorithms to assist health care providers in selecting the most appropriate contraceptive method for each patient. Updated in February 2025, it incorporates data on contraceptive costs in the United States, contraceptive adaptation, and natural contraceptive methods. This app uses a drop-down menu to aggregate static documents that cannot be navigated or interacted with.
- Practice Family Planning: this app was developed by the Global Health Media Project, a nonprofit organization specializing in the production of educational and preventive health videos. The app is available in French, English, and Spanish. The app offers approximately 40 videos tailored for both health care professionals and patients. Health care professionals can use them for self-training or to select and share with patients for information and education on sexual health and contraception. The videos were developed for developing countries or countries with special health conditions, but they can also be used in countries with a higher socioeconomic level. They teach professionals how to approach and present information on contraception. They also include educational videos for professionals on technical procedures such as intrauterine device insertion, implant insertion or removal, practice organization, and the prevention of nosocomial infections. The videos can be downloaded and viewed offline. This video directory is a useful tool to help professionals in their preventive actions, but some videos are of average quality and perform more reliably when downloaded locally.
- Learn Family Planning: this app, also developed by the Global Health Media Project, is very similar to the previous one. It offers videos to be shown directly to patients by professionals wishing to discuss contraceptive health. Developed for the same target audience as “Practice Family Planning,” the videos can also be used in countries with higher socioeconomic levels. The topics covered are more targeted than those in “Practice Family Planning,” as the videos are intended to be shared with patients: contraceptive methods, education on contraceptive use, method comparisons, pregnancy, and pelvic examinations.

## Discussion

### Main Results

This digital review of apps available on the Android and iOS platforms identified 976 apps addressing areas of prevention relevant to consultations between health care professionals and adolescents: vaccinations, orthopedics, drug use and addiction, mental health, nutrition, puberty, sexual health, social relations, and violence. Of these 976 apps, 7 (0.72%) were retained after analysis as recommendable to health care professionals for use in consultations to help them implement prevention measures with adolescents: 2 on mental health (including 1 sleep) and 5 on sexual health (including 3 contraception only). No apps specifically dedicated to adolescent prevention in any of the recommended areas were identified.

### Method Discussion

In the MARS-F, NA ratings are described in great detail for each item within the subscales [23]. Since NA ratings are not included in the final scores, these ratings could artificially inflate the scores and lead to the inclusion of apps of insufficient quality. In our study, NA ratings were found only in subsection D (Information), items 14, 17, and 19, which could increase this risk. However, the researchers were rigorous in their analyses, and this did not affect the selection of apps. We have analyzed these NA ratings found in the following:

- Item 14: Goals (Does the app have specific, measurable goals?). Only 2 apps (*Agenda du sommeil antifatigue* and *Bariatrique Québec*) received a numeric score on this item. The other 9 apps provide information but do not allow for any measurable goals.
- Item 17: Visual Information (NA=There is no visual information in the app; eg, it contains only audio or text). The NA rating is clear in this case.
- Item 19: Evidence Base (Scientific evidence: “Has the app been tested or evaluated, and is it supported by evidence in the published scientific literature?”). The NA is clear in this case. None of the apps has been evaluated in a published study. This item alone does not account for the scientific relevance of the app; item 15 (Quality of Information) supports the scientific quality of the information, as does the pre-evaluation screening of the apps (only apps that have been scientifically validated were included: inclusion criterion number 4).

### Safety, Liability, and Ethical Considerations

The integration of Clinical Decision Support Systems, such as the WHO Contraception Tool or mhGAP-IG 2.0 App, into practice carries significant medicolegal and ethical implications. These tools are designed to support, not replace, professional expertise. The health care professional remains solely liable for the final clinical decision. Most apps include disclaimers stating that recommendations are based on global guidelines and may not account for individual patient complexities. A critical risk is “automation bias,” in which a professional might overlook rare comorbidities or contraindications that are not programmed into the app’s algorithms.

A scoping review published in 2023 examined studies of apps used by health care professionals in clinical practice [37]. This scoping review aimed to describe how physicians use smartphones and mobile apps in clinical settings. Ten studies were included, mainly using survey or focus group methods. Smartphones and mobile apps were used for communication, medical education and training, clinical decision-making, and accessing drug compendia in most studies. Health care professionals were concerned about patient privacy and confidentiality. In our study, only 1 app (*Agenda du sommeil antifatigue*) involved personal data, but it was nonnominative and confidentiality was guaranteed. In addition, this app stands out as a robust model, being managed by the Bordeaux University Hospital, validated by a Data Protection Officer, and hosted within a secure institutional framework ensuring General Data Protection Regulation compliance. While other apps lack transparency regarding data encryption and remote storage, they do not collect or store any personally identifiable information or nominative data within the app.

The absence of age-verification and crisis-response features represents a limitation in adolescent-focused care apps. However, as the apps in our study are professional-facing tools, they lack autonomous safety triggers by design. Consequently, the safety of vulnerable users relies on practitioners’ expertise, with the clinical framework compensating for the absence of built-in safeguarding features.

### Comparison With the Literature

#### mHealth Apps

No study similar to ours, focusing on the use of apps to facilitate preventive action by health care professionals among adolescents, has been found in the literature. One review of apps used a method similar to ours but focused on patient-facing medication management apps [35]. The aim of this 2024 study was to identify and evaluate the medication management smartphone apps available in France that send reminders to patients. Sorting apps using the MARS-F grid enables a meticulous and stringent selection of recommended apps. Our study selected 7 apps out of 976, while that of Toïgo et al [35] selected 49 out of 960. The authors stated that the quality of the apps was heterogeneous, with only 2 having been studied in a randomized controlled trial with positive results. In our study, none of the apps had been the subject of a randomized controlled trial. Choosing which apps are relevant for health care professionals is difficult, as the scientific data used to develop these tools are often lacking, as are analyses of real-life use. By using a rigorous and reproducible selection process, we have identified and recommended the most effective apps for daily clinical practice, tailored for both patients and health care professionals.

The existing literature primarily comprises scientific studies evaluating apps designed for either health care professionals or patients, typically using systematic, narrative, or scoping review methodologies. A 2012 systematic literature review examined articles addressing the design, development, evaluation, or use of smartphone apps for health care professionals, medical or nursing students, or patients [38]. The authors retained data on 57 apps for health care professionals focusing on, among other things, disease diagnosis, drug reference, medical calculators, clinical communication, medical training, and general health care apps. The disease diagnosis, drug reference, and medical calculator apps were reported as the most useful by health care professionals.

Apps specifically designed to assist health care professionals with adolescent preventive care during clinical consultations were rare in our study (7/976). Our search for apps on the subject of adolescent prevention for health care professionals enabled us to identify apps that were relevant to health care professionals. Of the 91 apps that met the inclusion criteria, only 7 were retained as relevant to health care professionals. The other 82 were intended for adolescents or their parents. Adolescents are connected and use their smartphones for an average of 4 to 8 hours a day [39-42]. Apps addressing prevention themes that concern them are more likely to be developed for them than for health care professionals. Our findings confirm a significant gap: while thousands of apps exist for patient self-management, there is a striking lack of tools designed to support providers during consultations (Decision Support Systems). This reflects a general lack of investment in “Professional mHealth,” which is essential for preventive health care.

The small number of apps retained in our study was directly related to the specific inclusion and exclusion criteria established for our review. Notably, we chose to exclude paid apps to avoid imposing financial burdens on health care professionals and to preserve the absolute independence of our research, which is entirely free from conflicts of interest (ie, no recommendations of paid apps or of apps developed by the pharmaceutical industry). We would like all French and English-speaking health care professionals to be able to benefit, free of charge, from the results of our work. The exclusion criterion, which was also very selective, was the lack of explicit scientific backing for the development of an app. It was not appropriate to offer a health care professional a selection of apps that were not based on reliable scientific data. The MARS-F tool is widely used in the literature as a tool for assessing the quality of health apps: it is a simple, objective, and reliable tool for classifying and assessing the quality of mobile health apps [34,35]. The choice of this method for our work was, therefore, appropriate [34,43].

#### Many Areas of Prevention for Adolescents

There are many areas of prevention that need to be discussed with adolescents during a consultation with a health care professional. The development of apps designed to support health care professionals in their preventive work with adolescents does not always address current global public health challenges. Furthermore, the areas of prevention addressed by these digital tools are unevenly represented. A quantitative study carried out in France in 2021 confirmed that general practitioners mainly discussed orthopedics, sports activities, vaccinations, and contraception, but had more difficulty discussing the prevention of sexually transmitted infections or addiction when consulting an adolescent [7]. Two hundred eleven (21.6%) of the 976 apps were related to addictions and substance abuse, which could meet adolescents’ needs. No apps were selected because they had to be paid for or because they lacked scientific validation. The development of these apps may have been guided by a marketing and financial objective (paid apps) rather than by a public health interest (no scientific validation). General practitioners in the French 2021 study readily and spontaneously discussed orthopedics, vaccination, and contraception [7]. Of the 976 apps found in our screening, only 133 (13.6%) and 38 (3.9%) concerned orthopedics and vaccination. No apps were selected for health care professionals. As general practitioners are already involved in preventive work on these topics, the development of relevant and scientifically valid apps on these subjects was probably not a priority. No apps related to vaccination were selected. However, vaccination coverage among adolescents remains insufficient today. Despite the need for digital interventions on vaccination, high development costs and the necessity for frequent updates could represent significant barriers for developers. General practitioners need tools to discuss sexual health, but seem to find it easier to discuss contraception with adolescents. Only 41 apps about sexual health (4.2%) were identified out of the 976. At the final inclusion, sexual health apps accounted for 5 (71.7%) of the 7 apps selected as relevant for health care professionals. These 5 apps all deal with contraception, but other areas of sexual health are not covered, or only to a limited extent. This may be due to historical market trends, whereby sexual health was an early adopter of digital formats, as well as the ongoing need to improve adolescent reproductive health worldwide. Despite constant progress in contraception, the rate of unwanted pregnancies worldwide has changed little. Already in 2015, among 4793 sexually active American students, 488 (10.2%) said they only used the withdrawal method, and 594 (12.4%) used no contraceptive method at all [44]. Two Canadian studies in 2017 and 2019 found a prevalence of adolescent pregnancy ranging from 3.9% to 14.1%, depending on the group studied [45,46]. A 2024 French study entitled “The context of sexuality in France,” conducted from 2019 to 2023, reported some worrying results [47]. Among 18‐ to 29-year-olds, use of the pill fell from 54.3% to 36.6% between 2016 and 2023, use of the intrauterine device increased from 10.9% to 19.3%, but the absence of contraception rose from 4.3% to 8.7% [47]. The mental health of adolescents is a constant and indeed growing concern in France and around the world. This situation has worsened since the COVID-19 pandemic [48]. However, no mental health apps were included in our study. Although 370 (37.9%) of the 976 apps were related to mental health, no apps were selected because they had to be paid for or because they lacked scientific validation. Developers have adapted to the market and to the needs of health care professionals in relation to public health issues. However, the cost of the apps and ethical concerns about offering an app that has not been scientifically validated mean that no tool was selected.

### Implications for Policy and Practice

Analysis of the MARS-F subdomains reveals that the apps had higher scores in Information and Functionality (mean scores of 3.5 for both), while Engagement and Esthetics received lower ratings (mean scores of 2.0 and 2.5, respectively). This suggests that current apps are often developed as “digital brochures” rather than interactive tools. This situation is frequently observed in mHealth literature [49], where institution- or professional-facing apps prioritize clinical evidence and technical reliability over visual appeal or gamification. For a health care provider, a tool that is difficult to navigate during a consultation is unlikely to be adopted, regardless of the quality of its medical evidence. This implies the need for development frameworks. These frameworks have to integrate optimal user-centered design and esthetics to encourage users to engage with an app, so that tools can be more effective in clinical settings. Our findings align with the recent update by the High Authority for Health on the evaluation framework for mHealth apps [21]. The High Authority for Health confirmed that medical expertise and scientific evidence are mandatory for digital tools to be integrated into official health care pathways. However, our study shows that very few prevention-focused apps currently meet these high standards, particularly for the adolescent population. Medical societies and health authorities should establish certification labels or “official app libraries” to guide health care professionals. This would reduce the “search burden” for clinicians and ensure the quality of tools used with adolescents.

### Strengths and Limitations

#### Method

This is a systematic digital review, as all the apps were scanned to exhaustion during the initial 2 screenings. A solid methodology involving 7 researchers and the drafting of the study based on the PRISMA, PRISMA-S, and PASSR checklists reinforce the methodological rigor [24-26]. Finally, the use of a blind analysis based on a validated tool (MARS-F), combined with a detailed analysis of reliability, ensures the validity of the results by limiting selection bias.

Fifty-two apps disappeared during the course of the study. The ephemeral nature of the apps partly explains the small number of results and must be taken into account when sharing the results of our study, as with similar research projects. The disappearance of 52 apps during our study period highlights the high volatility of the digital health market, a major challenge for health care professionals seeking stable clinical tools and a structural limitation for any systematic digital review. Even though the selected apps successfully advanced through all screening stages, several of them still exhibit technical deficiencies that may negatively impact their clinical utility and usability. Although the strict exclusion criteria may have omitted certain apps with potential use for health care professionals, the fact that the selected apps are free, commercially independent, and scientifically validated significantly strengthens the clinical relevance and validity of our findings.

#### Reliability

A key strength of this study is the transparency regarding measurement error. By reporting the SEM and MAE alongside the ICC, we provide a realistic context for the quality scores. The fact that the SEM (0.60) is often larger than the score gaps between top-rated apps indicates that these tools are of comparable quality within the limits of the MARS-F scale’s precision. Since the ICC is a ratio of intersubject variance to total variance, the lack of variability between subjects in our small sample mathematically deflates the ICC, even when raters are in close agreement. The low ICC in some dimensions does not reflect poor rater performance but rather a “range restriction” effect, as the selected apps were homogeneously high in quality. This is confirmed by the good global MAE (0.53), indicating that, on average, raters differed by only half a point on a 5-point scale. This confirms that the ratings are clinically consistent and that the differences between the veteran expert and the residents represent an acceptable margin of measurement error rather than systematic disagreement.

The “Engagement” subscale had the highest statistical reliability (fair ICC=0.540 and excellent MAE=0.40), while the “Functionality” subscale exhibited the greatest rater divergence (poor MAE=1.03). Overall, the low absolute error across most dimensions confirms that the ratings provided by the residents are consistent with expert judgment for practical evaluation purposes.

While our rater calibration involved rigorous independent scoring and iterative consensus sessions, no formal statistical reliability metrics (such as a training ICC) were computed during this phase. This lack of a quantitative baseline check represents a limitation. It may explain why certain complex domains, specifically the Functionality subscale, demonstrated lower scoring homogeneity in the main evaluation, as reflected by an MAE of 1.03.

The global MARS-F score yielded an SEM of 0.60, providing essential context for comparing the evaluated apps. Given that the differences between the mean scores of several apps (eg, 3.5 vs 3.2) are smaller than this measurement error (Multimedia Appendix 2), this indicates that variations should be interpreted as qualitative trends rather than statistically significant differences in app quality (Table 4). The inclusion of the SD and range for each app further demonstrates the distribution of ratings and confirms that the observed consistency remains within acceptable clinical margins despite the small sample size.

#### Language

This study examines English and French apps evaluated by French health care providers. A selection was made to ensure the tools were applicable to clinical settings within these linguistic spheres. However, we acknowledge that perceptions of “clinical relevance” and “quality” are inherently shaped by national guidelines and cultural nuances. Consequently, while our findings offer robust insights for Western clinical contexts, they should be viewed as a foundation for further cross-cultural research across diverse linguistic and regulatory environments.

### Perspective

Our study conducted a rigorous selection of existing apps covering all areas of prevention recommended for adolescents during consultations with a health care professional. This type of review should be carried out systematically before any new tool is developed to avoid duplication and focus creative energy on innovative digital tools. Another study will look at existing websites that are relevant to helping health care professionals with their prevention work with adolescents during consultations. Similar to this study, it will examine websites in all the recommended areas of prevention, and the sites will be analyzed using a validated tool, Netscoring [50]. These 2 research projects (reviews of apps and websites) could be used as models for similar digital reviews prior to the creation of any new app or website. Finally, whatever tool is identified or designed, feasibility and acceptability studies by the professionals themselves will be necessary.

While the MARS-F scale offers a robust evaluation of information quality and credibility, it lacks explicit criteria for assessing clinical safety features. Future research should integrate specific safety frameworks to determine whether apps incorporate essential emergency protocols or risk-warning systems, particularly for users presenting with severe mental health symptoms.

Pending assessment of the acceptability and feasibility in clinical practice of the 7 apps selected in our study, we recommend 4 of these apps: “*Agenda du sommeil antifatigue*” and “mhGAP-IG 2.0 App” (with certain limitations linked to the technical shortcomings of “mgGAP-IG 2.0 App”); of the 5 sexual health apps, we recommend “WHO Contraception Tool,” available in several languages, and “Practice Family Planning,” which is richer in video content for health care professionals. These 5 apps could be included in the recommended tools for health care professionals on the planned global website.

To provide a comprehensive operational framework for the 4 recommended apps, Table 5 categorizes them based on the following 6 key implementation parameters:

- Clinical scenarios define the specific medical contexts (eg, diagnosis, prevention, or follow-up), where the app is most effective.
- Time-efficiency per consultation estimates the duration required to use the tool during a standard medical visit, ensuring that it remains compatible with professional time constraints.
- Technical information covers the hardware and software requirements (eg, operating system compatibility and offline access).
- Integration describes the clinical workflow: how the tool is incorporated into the patient-provider interaction or synchronized with health data management.
- Engagement refers to the strategies used to motivate health care professionals’ participation (eg, visual aids).
- Barriers to adoption include linguistic limitations, technical bugs, and cognitive load for practitioners.
- Mitigation strategies represent the proactive measures or professional safeguards proposed to minimize identified risks and overcome barriers, ensuring the safe and effective use of the digital tool within a supervised clinical framework.

**Table 5.** Guide for practical clinical implementation of the 4 recommended apps.

App name	Clinical scenarios and time efficiency per consultation	TI^a^ and integration	Engagement and barriers to implementation	Mitigation strategies
*Agenda du sommeil antifatigue*	Scenarios: Diagnosis of insomnia Sleep hygiene education Time: 5 min	TI: iOS (Apple) or Android (Google) French Integration: HCP^b^ reviews data entered by the patient	Engagement Active self-monitoring by the patient Barrier: Patient forgetfulness in logging data	The patient uses automated app notifications HCP validates data entry during each clinical visit
mhGAP-IG 2.0 App	Scenario: Assessment of mental or neurological disorders in nonspecialist settings Information on various neurological and mental disorders Time: 15 min (questionnaire and flowchart) or much more for information	TI: iOS or Android Multilanguage E-version of a WHO^c^ guide Integration: Decision support tool used during the clinical interview	Engagement: Standardized assessment tool Barrier: Some nonfunctional features	HCP should cross-reference with the physical or PDF WHO manual for nonfunctional app links
WHO Contraception Tool	Scenario: Selection of evidence-based contraception Checking for contraindications to current contraception Time: 3 min during the prescription phase	TI: iOS or Android Multilanguage Regularly updated Integration: Point-of-care tool to match medical history with contraception method	Engagement: Inclusion of patient preferences Barrier: Requires medical history data entry	Prepare a patient history checklist prior to app use to speed up data entry (patient or HCP)
Practice Family Planning	Scenarios: Professional self-training for women’s health consultations Clinical procedure preparation (eg, IUD^d^ insertion) Direct patient education on contraceptive health Time: 5‐15 min/video	TI: iOS or Android Multilanguage Offline mode available Integration: Preprocedural refresher for women health providers Shared screen education during consultation	Engagement: Visual learning Short videos Barrier: Average video quality Requires significant storage space	Preselect videos relevant to the practice of HCP Download videos prior to clinical sessions to ensure smooth playback offline

a TI: technical information.

b HCP: health care professional.

c WHO: World Health Organization.

d IUD: intrauterine device.

### Conclusions

There are many areas of prevention that need to be addressed with adolescents during consultations. Only 7 apps were relevant for helping health care professionals in their prevention work with adolescents. Five deal with sexual health, particularly contraception, and 2 with mental health. No apps have been validated for orthopedics, social relations, addictions, or vaccinations. Health care professionals are connected, especially online during consultations. A general online tool to help health care professionals with prevention among adolescents during consultations is relevant but currently nonexistent. Our study will contribute to the selection of relevant content for the development of such a tool, but further research is needed to ensure that it meets the needs of health care professionals.

## Supplementary material

10.2196/83908 Multimedia Appendix 1 Study protocol.

10.2196/83908 Multimedia Appendix 2 MARS-F scores for the 9 evaluated apps.

10.2196/83908 Multimedia Appendix 3 Abstracts of the 7 included apps.

10.2196/83908 Checklist 1 PRISMA checklist.

10.2196/83908 Checklist 2 PRISMA-S checklist.

10.2196/83908 Checklist 3 PASSR checklist

## Abbreviations

ICC: intraclass correlation coefficient
MAE: mean absolute error
MARS-F: French version of the Mobile App Rating Scale
mHealth: mobile health
NA: not attributable
PASSR: Protocol for App Store Systematic Reviews
PRISMA: Preferred Reporting Items for Systematic Reviews and Meta-Analyses
PRISMA-S: Preferred Reporting Items for Systematic Reviews and Meta-Analyses Literature Search Extension
SEM: standard error of measurement
WHO: World Health Organization

## References

[1] Sawyer SM, Azzopardi PS, Wickremarathne D, Patton GC (2018). The age of adolescence. Lancet Child Adolesc Health.

[2] da Conceição Taborda-Simões M (2005). L’adolescence: une transition, une crise ou un changement? [Article in French]. Bull Psychol.

[3] Basto-Pereira M, Miranda A, Ribeiro S, Maia Â (2016). Growing up with adversity: from juvenile justice involvement to criminal persistence and psychosocial problems in young adulthood. Child Abuse Negl.

[4] Peeters M, Oldehinkel A, Veenstra R, Vollebergh W (2019). Unique developmental trajectories of risk behaviors in adolescence and associated outcomes in young adulthood. PLoS One.

[5] Duell N, Steinberg L, Icenogle G (2018). Age patterns in risk taking across the world. J Youth Adolesc.

[6] Beck F, Richard JB (2013). Les Comportements de Santé Des Jeunes Analyses Du Baromètre Santé 2010 [Book in French].

[7] Vergne C, Bayard S, Rieu-Clotet L, Carbonnel F, Lognos B, Million E (2021). Which prevention fields are most frequently forgotten during consultations with adolescents and young adults? An ECOGEN ancillary cross-sectional study. Exercer.

[8] (2014). The Guide to Clinical Preventive Services 2014: Recommendations of the US Preventive Services Task Force.

[9] Alderman EM, Breuner CC, Committee on Adolescence (2019). Unique needs of the adolescent. Pediatrics.

[10] Yarnall KSH, Pollak KI, Østbye T, Krause KM, Michener JL (2003). Primary care: is there enough time for prevention?. Am J Public Health.

[11] Biglan A, Brennan PA, Foster SL, Holder HD (2004). Helping Adolescents at Risk: Prevention of Multiple Problem Behaviors.

[12] Binder P, Heintz AL, Tudrej B, Haller DM, Vanderkam P (2018). L’approche des adolescents en médecine générale Première partie. L’adolescent, cet inconnu [Article in French]. Exercer.

[13] Tudrej BV, Heintz AL, Ingrand P, Gicquel L, Binder P (2016). What do troubled adolescents expect from their GPs?. Eur J Gen Pract.

[14] Stheneur C, Alvin P, Boudaillez B (2009). La première consultation avec un adolescent [Article in French]. Arch Pediatr.

[15] Million E, Herbreteau M, Bourrel G (2025). Adolescents report insufficient preventive health care in GP consultations: a qualitative study. BJGP Open.

[16] du Roscoät E, Beck F (2013). Efficient interventions on suicide prevention: a literature review. Rev Epidemiol Sante Publique.

[17] Gelly J, Mentre F, Nougairede M, Duval X (2013). Preventive services recommendations for adults in primary care settings: agreement between Canada, France and the USA: a systematic review. Prev Med.

[18] (2009). Just between us: how to initiate and implement a health education approach with adolescents?. https://www.medecin-ado.org/addeo_content/documents_annexes/121-4-entrenousinpes.pdf.pdf.

[19] (2011). mHealth: new horizons for health through mobile technologies: second global survey on eHealth. https://iris.who.int/server/api/core/bitstreams/ad1b13c0-7c82-47b4-8dd5-f0a26c3a3cc3/content.

[20] Safon MO, Suhard V (2025). E-health: telehealth, digital health, or connected health. https://www.irdes.fr/documentation/syntheses/e-sante.pdf.

[21] (2021). Évaluation des applications dans le champ de la santé mobile (mhealth) - état des lieux et critères de qualité du contenu médical pour le référencement des services numériques dans l’espace numérique de santé et le bouquet de services des professionnels [Report in French]. https://www.has-sante.fr/upload/docs/application/pdf/2021-06/criteres_de_qualite_du_contenu_medical_referencement_mhealth_ens_2021-06-30_10-58-28_773.pdf.

[22] Della Vecchia C, Leroy T, Bauquier C (2022). Willingness of French general practitioners to prescribe mHealth apps and devices: quantitative study. JMIR Mhealth Uhealth.

[23] Saliasi I, Martinon P, Darlington E (2021). Promoting health via mHealth applications using a French version of the mobile app rating scale: adaptation and validation study. JMIR Mhealth Uhealth.

[24] Page MJ, McKenzie JE, Bossuyt PM (2021). The PRISMA 2020 statement: an updated guideline for reporting systematic reviews. PLoS Med.

[25] Rethlefsen ML, Kirtley S, Waffenschmidt S (2021). PRISMA-S: an extension to the PRISMA statement for reporting literature searches in systematic reviews. Syst Rev.

[26] Marshall JM, Dunstan DA, Bartik W (2020). Apps with maps—anxiety and depression mobile apps with evidence-based frameworks: systematic search of major app stores. JMIR Ment Health.

[27] Operating system market share worldwide. StatCounter Global Stats.

[28] (2026). Medical subject headings. National Library of Medicine (NLM).

[29] Google Help.

[30] Clarifying criteria & new timing extension for app store improvements process - latest news. Apple Developer.

[31] Crawford A, Serhal E (2020). Digital health equity and COVID-19: the innovation curve cannot reinforce the social gradient of health. J Med Internet Res.

[32] Krebs P, Duncan DT (2015). Health app use among US mobile phone owners: a national survey. JMIR Mhealth Uhealth.

[33] Folkvord F, Bol N, Stazi G, Peschke L, Lupiáñez-Villanueva F (2023). Preferences in the willingness to download an mHealth app: discrete choice experimental study in Spain, Germany, and the Netherlands. JMIR Form Res.

[34] Stoyanov SR, Hides L, Kavanagh DJ, Zelenko O, Tjondronegoro D, Mani M (2015). Mobile app rating scale: a new tool for assessing the quality of health mobile apps. JMIR Mhealth Uhealth.

[35] Toïgo M, Marc J, Hayot M, Moulis L, Carbonnel F (2024). Quality assessment of smartphone medication management apps in France: systematic search. JMIR Mhealth Uhealth.

[36] (2013). WMA Declaration of Helsinki – ethical principles for medical research involving human participants. World Medical Association.

[37] Lee M, Mahmood ABSB, Lee ES, Smith HE, Car LT (2023). Smartphone and mobile app use among physicians in clinical practice: scoping review. JMIR Mhealth Uhealth.

[38] Mosa ASM, Yoo I, Sheets L (2012). A systematic review of healthcare applications for smartphones. BMC Med Inform Decis Mak.

[39] Kosola S, Mörö S, Holopainen E (2024). Smartphone use and well-being of adolescent girls: a population-based study. Arch Dis Child.

[40] Bae SM (2017). Smartphone addiction of adolescents, not a smart choice. J Korean Med Sci.

[41] Kliesener T, Meigen C, Kiess W, Poulain T (2022). Associations between problematic smartphone use and behavioural difficulties, quality of life, and school performance among children and adolescents. BMC Psychiatry.

[42] Nagata JM, Al-Shoaibi AAA, Leong AW (2024). Screen time and mental health: a prospective analysis of the Adolescent Brain Cognitive Development (ABCD) Study. BMC Public Health.

[43] Salazar A, de Sola H, Failde I, Moral-Munoz JA (2018). Measuring the quality of mobile apps for the management of pain: systematic search and evaluation using the mobile app rating scale. JMIR Mhealth Uhealth.

[44] Liddon N, O’Malley Olsen E, Carter M, Hatfield-Timajchy K (2016). Withdrawal as pregnancy prevention and associated risk factors among US high school students: findings from the 2011 National Youth Risk Behavior Survey. Contraception.

[45] Fortin-Langelier E, Daigneault I, Achim J, Vézina-Gagnon P, Guérin V, Frappier JY (2019). A matched cohort study of the association between childhood sexual abuse and teenage pregnancy. J Adolesc Health.

[46] Wall-Wieler E, Roos LL, Nickel NC (2018). Adolescent pregnancy outcomes among sisters and mothers: a population-based retrospective cohort study using linkable administrative data. Public Health Rep.

[47] Andro A, Bajos N, Bellamine R, Bergström M, Beaubatie E (2024). Preliminary results of the national survey “Context of Sexualities in France 2023”. ANRS Maladies infectieuses émergentes (ANRS MIE).

[48] Jones EAK, Mitra AK, Bhuiyan AR (2021). Impact of COVID-19 on mental health in adolescents: a systematic review. Int J Environ Res Public Health.

[49] Baumel A, Muench F, Edan S, Kane JM (2019). Objective user engagement with mental health apps: systematic search and panel-based usage analysis. J Med Internet Res.

[1] D Darmoni SJ, Leroux V, Thirion B, Santamaria P, Gea M (1999). Net Scoring ®: critères de qualité de l’information de santé sur l’Internet [Article in French].

