# Supplementary material for: Smartphone Apps for Preventing Adolescent Health Problems Among Health Care Professionals: Systematic Search and Quality Assessment
Source: JMIR Mhealth Uhealth. 2026 Sep 11;14:e83908. doi: 10.2196/83908 (PMC13568167; doi:10.2196/83908)
Supplement: Supplementary file 1 [file mhealth-v14-e83908-s001.docx]

# Study Design and guidelines

## Study Design:

A systematic review of smartphone apps with an analysis of content quality was carried out by 4 researchers. 1 GP resident (CB) and 1 senior GP (EM) carried out the search of apps on the search engines and the first selection. 2 GP residents (MK and LP) and 1 senior GP (EM) evaluated apps using the French Version of the Mobile App Rating Scale (MARS-F) [1]. The study protocol was design following the PRISMA (Preferred Reporting Items for Systematic Reviews and Meta-Analyses) checklist (items not relevant to a systematic search of apps were considered not applicable) (multimedia supplementary 1) And PRISMA-F Checklist [2,3] (We have taken into account the specialized reporting items suggested by the PASSR (Protocol for App Store Systematic Reviews) checklist to address the unique challenges of searching and analyzing mobile application stores [4].

## MARS-F:

The MARS-F scale assesses four objective dimensions: Engagement, Functionality, Aesthetics, and Information quality. A subjective quality score and an app-specific section could also be completed. The tool and its French version (MARS-F) have been validated for the overall quality assessment of an app in the field of mHealth [1,5,6]. MARS-F is a 19-item questionnaire divided into 4 objective evaluation sections (A to D): engagement (5 items: entertainment, interest, personalization, interactivity, and adaptation to target group), functionality (4 items: app performance, ease of use, navigation, and app gesture design), aesthetics (3 items: layout, graphic design, and overall visual appeal), and information (7 items: accuracy of app description, precise app objectives, quality of information, quantity of information, visual information, credibility of information, and scientific evidence (Multimedia supplementary 3). The 5th section or Section E is a subjective assessment of the quality of the application. avec 4 questions : « Would you recommend this app to people who might benefit from it?»; « How many times do you think you would use this app in the next 12 months if it was relevant to you?»; « Would you pay for this app? »; « What is your overall star rating of the app? » from 1 to 5 stars. We have not retained this section E in the analysis of apps. Finally, Section F of MARS-F assessed intentions to change with the application: this was not the focus of our study, so we have not retained it for the evaluation of apps.

Each item is rated with a Likert scale from 1 (“inadequate”) to 5 (“excellent”). When specific objectives were not delineated, and information was lacking, the respective items were rated as “not applicable” (NA).Consequently, these items were excluded from consideration in the overall scoring process. In total, MARS-F is a score out of 5, corresponding to the average of these 4 sections. An average for each section from 1 to 5 was calculated for sections A, B, C and D for each evaluator, followed by an average for each inter-evaluator section. An overall average of the inter-rater MARS-F score was used to evaluate the apps. MARS-F required an in-depth analysis of the application, which revealed the presence of exclusion criteria that had not been identified during the selection process; it was in this context that the MARS-F exclusion criteria were selected. During the Quality appraisal, if an exclusion criterion was found by only 1 of the 2 evaluators for the final evaluation score, the 3rd researcher was asked to discuss the results and a consensus was sought.

# Eligibility criteria

## General eligibility criteria

The selection of apps was made on the basis of exclusion and inclusion criteria from digital platforms. To meet our inclusion criteria, the apps had to (1) relate to one of the selected areas of prevention (Orthopedics, mental health, substance abuse, risk behaviors, sexual health, vaccinations, social relations, nutrition ); (2) be relevant in facilitating the preventive approach of the health professional in consultation with adolescent; (3) be available in French or English ; (4) have been scientifically validated (developed by healthcare professionals or a medical learned society and/or based on scientific or government recommendations); (5) be free (apps free for only a trial period were not selected); (6) updated within the last 3 years before the end of the search at least by 2020 (since 2022, Google Play Store removes apps without updates for more than 2 years [7], and for the App Store, this period is 3 years for apps with few downloads in the last 12 months [8] but not all catalogues have yet been updated.

The exclusion criteria were: (1) no information concerning the scientific data used to design the content of the application; (2) games with no medico-educational action; (3) wallpaper apps; (4) religious apps; (5) apps offering links to medical appointments; (6) advertising apps. We have decided to keep only free apps, as healthcare professionals have already invested in costly practice tools such as business software and telecare tools. We therefore wanted to offer them apps that had no impact on their professional workloads, so that they would not be reluctant to use them. The high cost of an app is one of the main reasons why it is not downloaded [9,10].

## MARS-F eligibility Criteria

To ensure the selected applications were suitable for clinical practice and met the needs of healthcare professionals working with adolescents, specific quality cut-off scores were applied during the screening phase. Applications were excluded if they failed to meet the following minimum thresholds across the MARS-F sections:

- Target Group (Section A - Engagement): Apps were excluded if Item 5 (Target group) scored below 3, ensuring the content was appropriately tailored to the intended audience (Healthcare professional).
- Functionality (Section B): A minimum score of 2 was required for all items in this section to guarantee basic technical usability in clinical settings.
- Information Quality (Section D): Strict criteria were applied to ensure scientific relevance. Apps were excluded if Item 15 (Quality of Information) was rated NA or below 2, if Item 16 (Quantity of Information) was below 2, or if Item 17 (Visual Information) scored below 4. An app that provided very low information scores or had missing data was not recommended for healthcare professionals.

This rigorous selection process ensured that only tools with sufficient technical and informational integrity were included in the final analysis.

# Information sources

The Apple App Store and the Google Play Store where used for the systematic search.

As it was an app review, cited references were examined only to confirm scientific validity of the app.

We don’t contact authors, experts, manufacturers, or others.

# Search strategy

## Full search strategies

A systematic search was performed on the Apple App Store and the Google Play Store. CB and EM carried out the search on an IPhone 11* smartphone with the iOS 15 update, an ASUS VivoBook* computer and a Google Pixel 7* smartphone with the S91xBXXS1AWD1 update.

The platforms were queried in French and English using keywords related to adolescent prevention areas derived from Mesh words (Medical Subject Headings) [11] pre-identified in a literature review according to prevention themes (table 1). Mesh words were changed to a non-Mesh synonym when the search did not find any relevant data. Each keyword was searched for separately on the 2 digital platforms. The final choice of Mesh words was discussed between the 2 researchers CB and EM.

Table 1: English Key Words

| Prevention areas | English key words |
| --- | --- |
| Orthopedics | Kyphosis  Spinal curvatures  Vertebral Body  Lordosis  Spinal or vertebral column  Scoliosis  Back pain  Osgood-Schlatter disease^a^  Osteochondrosis  Scheuermann disease^a^  patellofemoral pain syndrome |
| Substance abuse, screens and addictions | Alcohol  Nicotiana  Drug  Cannabis  Heroin prevention^b^  Cocaine  Ecstasy  Screen Time |
| Mental health | Sleep Wake Disorders  Anxiety  Depression  Suicide  ADDH  Psychosis  Autism |
| Nutrition | Obesity  Deficiency diseases  Feeding and Eating Disorders |
| Sexual health and puberty | Sexual Transmitted Infection  Unsafe sex  Contraception  Puberty |
| Social relations and violence | School dropout  Harassment, Bullying^a^  Friend  Violence |
| Vaccinations | Vaccination |

^a^ Non-Mesh words

^b^The term "heroin" was not retained because the search yielded only games

## Limits and restrictions

2 similar researches, with the same method, was conducted between August 2022 and March 2023 and then between March and May 2023. Each keyword search was conducted on the same day. All keywords were entered into the digital platform’s search function one after another, without a specific pre-determined date.

We chose French terms because we need to reach French speaking professionals and English terms as it is a language understood by the entire healthcare community.

## Search filters

## No filters were used on the platforms. Each keyword was used separately. All of the results have been analyzed.

## Prior work

## No review of the apps was conducted prior to this study.

## Updates and dates of searches

To update the research 2 similar researches, with the same method, was conducted between August 2022 and March 2023 and then between March and May 2023. The apps evaluations with MARS-F took place between December 2023 and January 2024. At each stage of the study, the selected apps were reopened on the smartphones to ensure they remained installed. Some apps might have been deleted before the end of the study.

# Selection process and data collection process

The selection (EM, CB) followed a two-stage process First EM and CB screened the apps based on their titles, descriptions, and metadata provided in the stores. Second, the shortlisted apps were downloaded and tested for full eligibility.

The steps involved in selecting apps for each keyword were as follows:

1- Search by keyword using each digital platform's search engine
2- Read the app's title
3- For apps whose titles were related to the search keyword: the app description was opened on the platform. Apps whose titles were not informative or raised doubts about whether the content matched the search keyword were also opened.
4- Review the app description and include the app if there are no exclusion criteria listed in the description. In case of doubt about an exclusion criterion, the decision was made during discussions between CB and EM.
5- Information for each app’s dashboard: app name, digital platform, keywords used.
6- Repeat steps 2 through 5 for each app listed by the digital platform based on a keyword until all results have been exhausted, with no limit on the number of apps.
7- Systematically check whether the apps included on one digital platform were also available on the second platform to determine whether they were compatible with Apple smartphones, Android smartphones, or both.
8- From the dashboard, open the apps selected by the two researchers (EM and CB) in a double-blind manner and fill in the additional items in the table: for healthcare professionals, for teenagers or their parents, free of charge, scientifically validated content. At this stage, a discussion between the two researchers was necessary for : apps for which doubts remained regarding the presence of an exclusion criterion after reading the digital platform’s description, and apps whose target audience was not immediately clear upon first reading.
9- Excluding apps: intended exclusively for teenagers or their parents, either paid or containing paid content, with no information regarding the scientific validity of the content, unavailability on download platforms at the time of evaluation by one of the evaluators.

# Data analyzing

### Quality appraisal and data extraction:

From the apps selected in digital platforms by EM and CB, 3 researchers took part in evaluating the apps: 2 GP residents (MK and LP) divided up the prevention themes for analysis and 1 senior GP (EM) blindly analyzed all the apps. The all 3 used the French Version of the Mobile App Rating Scale (MARS-F) [1] filled separately. An individual table was filled then a common table in based on these MARS-F.

## Residents training:

Prior to beginning the evaluation, residents received training from the senior GP. 2 apps were used for this training. This step helped to informal reliability check during calibration. The following steps were followed for this training using two apps selected at random from the listed applications.

1- First working meeting (2 hours) of the app analysis team (EM, MK, LP): joint review of the MARS-F questionnaire and the instructions for completing it. Discussion with EM (senior GP) to ensure a clear understanding of all items and the methodological choices made for the study protocol (exclusion of sections E and F).
2- Separate, blinded analysis of each of the two applications by the three researchers, who each independently completed a MARS-F item rating table. Note in the margin of the table listing the issues raised by the analysis and the uncertainties in the valuations
3- Compilation of ratings in a shared spreadsheet and comments prior to the second meeting among the three researchers.
4- Second working meeting (2 hours) among the three researchers 3 weeks after the fist one. 1^st^ app : Review of each quote individually and discussion of discrepancies. Review of the criteria and instructions for completing the form. Automatically correct the criteria for the second app separately. 2^nd^ app : Review of each quote individually and discussion of discrepancies. Review of the criteria and instructions for completing the form.
5- Following the meeting, EM considered their understanding of the analytical method to be complete.
6- Throughout the formal evaluation period, raters had access to expert guidance to resolve technical queries. Specific applications were not discussed until the final consensus meeting to maintain blinded assessment integrity.
7- While a formal training ICC was not calculated, this iterative calibration was designed to minimize subjective bias.

## App blinded analyze:

After the training session, app blinded analyses were conducted, with scores for each item reviewed and discussed collectively at the end of the complete analyzis:

1- Breakdown of app analysis by theme between MK and LP.
2- Analysis of the apps by MK and LP based on their themes and completion of the MARS-F table.
3- At the same time, a blind analysis of all apps by EM.
4- Sharing information during a joint meeting. Discussion of discrepancies and corrections as needed, using the item descriptions from the MARS-F.

## Statistical Analysis

Mars-F scores are presented as means and standard deviations (SD).To assess the reliability of the MARS-F ratings (Global and subscales), we employed a multi-metric approach. Inter-rater reliability was calculated using the Intraclass Correlation Coefficient (ICC), model (2,1) (two-way random effects, absolute agreement, single measures), with the SD. To address measurement precision, we calculated the Standard Error of Measurement (SEM) using the formula SEM = SD × √(1 – ICC). Additionally, the Mean Absolute Error (MAE) was computed to provide an intuitive measure of the average point difference between raters. Descriptive statistics, including the mean and standard deviation (SD), were generated for each application and for each global scale or subscale to characterize the distribution of quality ratings. We analyzed the senior expert's ratings (EM) against the residents' ratings (MK/LP) [12].

An ICC above 0.75 was considered excellent, good between 0.60-0.74, fair between 0.40-0.59 and poor if below 0.40. Since the ICC is a ratio of inter-subject variance to total variance, the lack of variability between subjects in our small sample could mathematically deflates the ICC, even when raters are in close agreement. To provide a more accurate assessment of rater reliability, we have also calculated the Mean Absolute Error (MAE). The MAE represents the actual average point difference between raters on the 5-point scale. We have applied the following interpretation thresholds for MAE: Excellent (<0.5), Good (0.5–0.75), Fair (0.75–1.0), and Poor (>1.0).

ICC were performed with JASP (version 0.96; 2026; University of Amsterdam), MAE, SD, Mean and SEM vas calculated with Excel tool.

# references

1.	Saliasi I, Martinon P, Darlington E, Smentek C, Tardivo D, Bourgeois D, Dussart C, Carrouel F, Fraticelli L. Promoting Health via mHealth Applications Using a French Version of the Mobile App Rating Scale: Adaptation and Validation Study. JMIR Mhealth Uhealth 2021 Aug 31;9(8):e30480. PMID:34463623

2.	Page MJ, McKenzie JE, Bossuyt PM, Boutron I, Hoffmann TC, Mulrow CD, Shamseer L, Tetzlaff JM, Akl EA, Brennan SE, Chou R, Glanville J, Grimshaw JM, Hróbjartsson A, Lalu MM, Li T, Loder EW, Mayo-Wilson E, McDonald S, McGuinness LA, Stewart LA, Thomas J, Tricco AC, Welch VA, Whiting P, Moher D. The PRISMA 2020 statement: An updated guideline for reporting systematic reviews. PLoS Med 2021 Mar;18(3):e1003583. PMID:33780438

3.	Rethlefsen ML, Kirtley S, Waffenschmidt S, Ayala AP, Moher D, Page MJ, Koffel JB, PRISMA-S Group. PRISMA-S: an extension to the PRISMA Statement for Reporting Literature Searches in Systematic Reviews. Syst Rev 2021 Jan 26;10(1):39. PMID:33499930

4.	Mehrotra S, Tripathi R, Sengupta P, Karishiddimath A, Francis A, Sharma P, Sudhir P, Srikanth TK, Rao G, Sagar R. Evaluating Characteristics and Quality of Mental Health Apps Available in App Stores for Indian Users: Systematic App Search and Review. JMIR mHealth and uHealth JMIR Publications Inc., Toronto, Canada; 2025 Sept 26;13(1):e79238. doi: 10.2196/79238

5.	Stoyanov SR, Hides L, Kavanagh DJ, Zelenko O, Tjondronegoro D, Mani M. Mobile App Rating Scale: A New Tool for Assessing the Quality of Health Mobile Apps. JMIR mHealth and uHealth JMIR Publications Inc., Toronto, Canada; 2015 Mar 11;3(1):e3422. doi: 10.2196/mhealth.3422

6.	Toïgo M, Marc J, Hayot M, Moulis L, Carbonnel F. Quality Assessment of Smartphone Medication Management Apps in France: Systematic Search. JMIR Mhealth Uhealth 2024 Mar 18;12:e54866. PMID:38498042

7.	Developer Program Policy - Play Console Help. Available from: https://support.google.com/googleplay/android-developer/answer/16070163?hl=en [accessed Apr 22, 2025]

8.	Inc A. Clarifying criteria & new timing extension for App Store Improvements process - Latest News - Apple Developer. Available from: https://developer.apple.com/news/?id=gi6npkmf [accessed Apr 22, 2025]

9.	Krebs P, Duncan DT. Health App Use Among US Mobile Phone Owners: A National Survey. JMIR Mhealth Uhealth 2015 Nov 4;3(4):e101. PMID:26537656

10.	Folkvord F, Bol N, Stazi G, Peschke L, Lupiáñez-Villanueva F. Preferences in the Willingness to Download an mHealth App: Discrete Choice Experimental Study in Spain, Germany, and the Netherlands. JMIR Form Res 2023 Dec 25;7:e48335. PMID:38145470

11.	Mots clé MeSH et CISMeF | Département Médecine Générale - Université Paris Cité. Available from: https://dmg-u-paris.fr/p/mots-cle-mesh-et-cismef [accessed Apr 22, 2025]

12.	Stoyanov SR, Hides L, Kavanagh DJ, Zelenko O, Tjondronegoro D, Mani M. Mobile App Rating Scale: A New Tool for Assessing the Quality of Health Mobile Apps. JMIR mHealth and uHealth JMIR Publications Inc., Toronto, Canada; 2015 Mar 11;3(1):e3422. doi: 10.2196/mhealth.3422
