# Supplementary material for: Smartphone Apps for Preventing Adolescent Health Problems Among Health Care Professionals: Systematic Search and Quality Assessment
Source: JMIR Mhealth Uhealth. 2026 Sep 11;14:e83908. doi: 10.2196/83908 (PMC13568167; doi:10.2196/83908)
Supplement: Supplementary file 2 [file mhealth-v14-e83908-s003.docx]

This is a Multimedia Appendix to a full manuscript published in the JMIR Mhealth Uhealth. For full copyright and citation information see http://dx.doi.org/10.2196/jmir.xxxx” (where xxxx is your JMIR manuscript number – eg, http://dx.doi.org/10.2196/jmir.931 - note that this link only works *after* publication).

1- **Agenda du sommeil antifatigue**

This French-language application enables patients to fill in their "sleep diary" to gain a better understanding of their habits and sleep efficiency, and potentially improve their sleep hygiene. The application is also useful for health professionals, enabling them to objectivize the teenager's sleep habits and identify any sleep difficulties. The health professional can then use it to optimize his or her management of sleep disorders.

**Title:** Agenda du sommeil anti fatigue

**Authors:** Pr Pierre Philip and other physicians/researchers in the research unit CNRD UMR 6033 SANPSY du CHU et l’Université de Bordeaux.

**Ios and/or Android :** Ios and Android

**Developer :**  Bordeaux Hospital and University

**Language :** French

**Scientific Committee:** Université de Bordeaux and Pr Pierre Philip

**Data protection:** Health and usage data collected but no link established with user identity

**Release date:**  2020

**Update:**  2022

**Target group:** General population and physicians

**Objectives:** Record your sleep and evaluate its effectiveness

**Features:** Sleep diary: the patient notes his or her bedtime, the time he or she falls asleep, wakes up, gets up, the number of times he or she wakes up during the night and the quality of his or her sleep/wakefulness. The application calculates sleep efficiency. No other functions.

**Design, usability :** Easy handling thanks to simple functionality. Basic aesthetics.

**Remarks :** App developed as part of an "anti-fatigue" therapeutic education program to manage fatigue and related lifestyle habits.

2- **mhGAP-IG 2.0 app**

This application, available in several languages including English and French, is designed for care providers worldwide, based on WHO recommendations. It is the electronic version of the WHO mhGAP intervention guide for tackling mental, neurological and substance use disorders in non-specialized care settings. The application provides information on mental, neurological and substance-related disorders. It offers detailed descriptions of the diseases, questionnaires for identification and/or assessment, and recommendations for treatment. This application is particularly useful for doctors, enabling them to identify mental disorders and self-aggressive behavior in adolescents. Despite the "non-excluded" MARS criteria, there are a number of non-usable features that impact on the use of the tool.

**Titre :** mhGAP-IG 2.0 app

**Authors :** WHO

**Ios and/or Android :** Ios and Android

**Developer :** Universal Projects and Tools S.L.

**Language:** French, English, Spanish, Turkish and Ukrainian.

**Scientific Committee:** WHO with numerous psychiatric experts contributing to the drafting of the mhGAP-IG 2.0

**Data protection :** no personal data

**Release date :** 2017

**Update :** 2021

**Target group:** Health professionals

**Objectives :** The aim is to improve care for people with mental disorders (depression, psychosis, dementia, epilepsy, mental disorders/behavioral disorders in children and adolescents, self-aggressive/suicidal behavior, substance use disorders, other pervasive psychological complaints) in non-specialized care structures.

**Features :** Each module comprises 4 sub-categories: description, assessment, management and follow-up. Depending on the answers given in the assessment section, the professional is directed to different tabs in the management section.

**Design, usability :** Sober yet intuitive design. Easy to use.

**Remarks :** This application is the digital version of the mhGAP-IG, a simple technical tool using clinical decision-making protocols for referenced mental disorders. Some functionalities do not function optimally or systematically: assessment algorithm, management details. It is still possible to complete the guide by clicking on the pdf link.

3- **Who Contraception Tool**

This application, developed by the WHO, is developed to help healthcare professionals select the contraception that is best suited to the patient's medical history and characteristics and that complies with good practice recommendations. Contraceptive methods are ranked from 1 to 4 according to the level of recommendation (1: use possible, 2: use possible with conditions, 3 and 4: use not recommended). This application use the WHO 2015 recommendations and is available in several languages including English and French. Professional can also be used to add patient preferences, and provides information on emergency contraception, contraceptive efficacy and antiretroviral drugs.

**Title:** WHO contraception tool

**Author:** WHO

**Ios and/or Android :** Ios only

**Developer** : RC DIGITAL LTD OOD

**Languages :** French, English, Burmese, Spanish

**Scientific Committee:**  WHO

application based on the WHO publication "medical eligibility criteria for contraceptive use", 5th edition, 2015.

**Data protection:** Health and usage data collected but no link established with user identity

**Release date:**  2020

**Update :**  2024

**Target group:** health professionals

**Objectives:** Select the right contraception for women, taking into account their biological data and history, and taking into account their preferences.

**Features:** The patient's characteristics are entered into the algorithm, which ranks contraceptive methods according to possibility of use or contraindications from 1 to 4 (1: use possible, 2: use possible with conditions, 3: use not recommended, 4: contraindicated). The woman's preferences can be added to the algorithm (STI prevention, patient-controlled, regular bleeding, non-hormonal, privacy (discreet methods), long duration, etc.). A comparison of the effectiveness of contraceptive methods is also proposed. A list of antiretroviral treatments is provided, with possible interactions with certain contraceptive methods.

**Design, usability :** Extremely easy to handle, with a clear, helpful design for professionals (color codes, icons, etc.). A "how to use this tool" help page makes it easy to get started.

**Remarks :** No

4- **Contraception Eligibility Tool**

This app is the exclusive English version of the "Who Contraception Tool" app but based on 2 WHO recommendations from 2015 and 2018. It has been developed for humanitarian professionals. It is a tool for selecting recommended contraception choices based on patients' medical data and history, with the same 1 to 4 ranking key as the previous 2 apps. Additional information is available on initiating contraception, infertility, emergency contraception, method effectiveness, violence against women and managing forgotten pills.

**Title :** contraception eligibility tool

**Authors:** WHO

**Ios and/or Android :** Ios only

**Developer** : RC DIGITAL LTD OOD

**Language :** English

**Scientific Committee:** WHO

application based on WHO "medical eligibility criteria for contraceptive use", 5th edition, 2015 and WHO "selected practice recommendations for contraceptive use", 3rd edition, 2016

**Data protection:** Health and usage data collected but no link established with user identity

**Release date:**  2018

**Update :**  2020

**Target group :** Health professionals

**Objectives :** Select the right contraception for women, taking into account their biological data and history, and taking into account their preferences. Learn about emergency contraception and violence against women.

**Features:** The patient's characteristics are entered into the algorithm, which ranks contraceptive methods according to possibility of use or contraindications from 1 to 4 (1: use possible, 2: use possible with conditions, 3: use not recommended, 4: contraindicated). A comparison of the efficacy of contraceptive methods, recommendations for initiating contraception and emergency contraception, and information on violence against women are also available.

**Design, usability :** This application is less intuitive and less colorful in design than the international "WHO contraception tool". The "how to use the tool" section explains how to interpret the ranking of contraception methods. A "how to use the tool" help page makes it easy to get started.

**Remarks :** No

5- **Contraception Point-of-care**

This application, in English, offers algorithms for choosing the contraception best suited to the patient. Recently updated in February 2025, it also addresses the cost of contraception in the U.S., contraceptive adaptation and natural contraceptive methods. This application groups together documents from a drop-down menu, with no navigation or interaction possible on the application.

**Title :** Contraception point-of-care

**Authors :** Joshia Steinberg MD, Katherine Holmes

**Ios and/or Android :** Ios only

**Developer** : United Health Services, Inc.

**Language :** English

**Scientific Committee:** RHAP, UHS Wilson Family Medicine Residency faculty clinician and Dr Joshua Steinberg

application based on WHO "medical eligibility criteria for contraceptive use", 5th edition, 2015 and WHO "selected practice recommendations for contraceptive use", 3rd edition, 2016, US recommendations from the Reproductive health Access Project (RHAP) updated in 2025

**Data protection:** Health data (periods) but anonymous

**Release date :**  2017

**Update :** 2025

**Target group:** health professionals

**Objectives :** help with contraceptive prescriptions (side effects, contraindications, changes in treatment...)

**Features:** only informative, with a drop-down menu, without interactive functionality

**Design, usability:** basic design, no interaction possible. possibility of creating a calendar rules, source websites cited but without hyperlinks

**Remarks** : almost no interactivity

6- **Practice Family Planning**

This application is the brainchild of the Global Health Media Project, a non-profit organization specializing in the production of educational and preventive health videos. The application is available in French, English and Spanish. The application features some forty videos aimed at healthcare professionals and patients. Healthcare professionals can use them for self-training, or select them to share with patients for information or education on sexual health and contraception. The videos were developed for developing countries or countries with special health conditions, but can also be used in countries with higher socio-economic levels. They teach professionals how to approach and present information on contraception. They also include instructional videos on technical procedures such as IUD insertion, implant insertion and removal, office organization and prevention of nosocomial infections. The videos can be downloaded and consulted off-line. This video directory is a useful tool to help the professional in his or her preventive actions, but some videos are of average quality and work best if downloaded.

**Title :** Practice Family Planning

**Authors :** Global Health Media Project, in particular Eric Zhang et Elon Danziger

**Ios and/or Android :** Ios only

**Developer** : Global Health Media Project

**Language:** French, English, Spanish

**Scientific Committee:** Global Health Media Project

**Data protection:** None use of personal data

**Release date:**  2022

**Update :**  2023

**Target group :** Health professionals

**Objectives :** Select the best contraceptive method for each woman, learn about technical procedures (IUD insertion, pelvic examination, etc.).

**Features :** 40 videos available (languages: French, English, Spanish) on 5 main topics: contraceptive methods, advice on applying contraception (IUDs, implants, etc.), prevention of infectious risks. Videos can be saved for offline viewing, and sent to someone via Messenger / Instagram / message / airdrop ...

Donation link in the basic menu.

**Design, usability :** Rather old-looking videos, not always in sharp focus, occasional latency when launching videos. But clear, educational content

**Remarks** : Application developed for developing countries. Link to a donation in the "about Practice Family Planning" page.

7- **Learn Family planning**

This application, also a product of the Global Health Media Project, is very similar to the previous one. It offers videos to be shown directly to patients by professionals wishing to discuss contraceptive health. Developed for the same target audience as "practice family planning", the videos can also be used in countries with a higher socio-economic level. The topics covered are more targeted than those covered by "practice family planning", as the videos are intended to be shared with patients: contraceptive methods, education in contraceptive use, comparison of methods, pregnancy and pelvic examination.

**Title :** Learn Family planning

**Authors :** Global Health Media Project, in particular Eric Zhang et Elon Danziger

**Ios and/or Android :** Ios only

**Auteurs :** Global Health Media Project, in particular Eric Zhang et Elon Danziger

**Ios et/ou Android :** Ios

**Developer** : Global Health Media Project

**Language :** French, English, Spanish

**Scientific Committee:** Global Health Media Project

**Data protection:** no use of personal data

**Release date:**  2022

**Update :**  2023

**Target group:** Health professionals

**Objectives :** Select the best contraceptive method for each woman, learn about technical procedures (IUD insertion, pelvic examination, etc.).

**Features:** 24 videos available (languages: French, English, Spanish) on 5 main topics: contraceptive methods, advice on applying contraception (IUDs, implants, etc.), prevention of infectious risks, pregnancy. Videos can be saved for offline viewing, and sent to someone via Messenger / Instagram / message / airdrop ...

Donation link in the basic menu.

**Design, usability :** Rather old-looking videos, not always in sharp focus, occasional latency when launching videos. But clear, educational content.

**Remarks** : Very little interactivity; videos are of higher quality if downloaded in advance. The app was developed for developing countries. Link to a donation on the "About Practice Family Planning" page.
